# Shexiang Baoxin Pills Inhibited Proliferation and Migration of Human Coronary Artery Smooth Muscle Cells *via* PI3K/AKT/mTOR Pathway

**DOI:** 10.3389/fcvm.2021.700630

**Published:** 2021-08-25

**Authors:** Lei Hua, Yaqing Zhou, Can Hou, Jiaxin Chen, Yanjun Wang, Sheng Zhang, Hanxiao Zhou, Shu He, Enzhi Jia

**Affiliations:** Department of Cardiovascular Medicine, The First Affiliated Hospital of Nanjing Medical University, Nanjing, China

**Keywords:** coronary artery disease, smooth muscle cell, traditional Chinese medicine, network pharmacology, proliferation and migration

## Abstract

**Background:** Proliferation and migration of smooth muscle cells in the coronary artery contribute to the deterioration of coronary artery disease (CAD).

**Aim:** This research was designed to study the function of Shexiang Baoxin pills (SBPs) on the proliferation and migration of human coronary artery smooth muscle cells (HCASMCs) and their mechanism.

**Methods:** Oxidized low-density lipoprotein (ox-LDL) was applied to stimulate the proliferation and migration of HCASMCs. The function of ox-LDL and SBP on HCASMCs was evidenced by the cell counting kit-8 assay, cell cycle, and Transwell assay. Network pharmacology was employed to predict the potential targets and pathways of SBP on CAD. Western blot assay and molecular docking were conducted to validate the potential targets and pathways.

**Results:** The current research revealed that 2.5 mg/L SBP significantly inhibited the proliferation and migration of HCASMCs. Besides, network pharmacology revealed 11 candidate targets. Molecular docking and Western blot assay validated that the activation of the top 2 targets STAT3 and MAPK14 was associated with the inhibition of HCASMCs. Moreover, the Western blot assay also detected that HCASMCs treated with ox-LDL promoted the phosphorylation of the PI3K/AKT/mTOR pathway, and SBP inhibited the activation of the PI3K/AKT/mTOR pathway in HCASMCs stimulated by ox-LDL.

**Conclusion:** This study demonstrated that the treatment of CAD using SBP may result from the suppression of the proliferation and migration of HCASMCs. The mechanism of this function partly resulted from relieving the phosphorylation of targets STAT3 and MAPK14 and the PI3K/AKT/mTOR pathway. This study enhanced our comprehension of SBP and provides new targets for the treatment of CAD.

## Introduction

Coronary artery disease (CAD) remains the leading cause of mortality worldwide. Despite prevention strategies and medical treatment advances, the prevalence of CAD has increased among developing countries (1–3). A major characteristic of atherosclerotic plaque development is the migration of vascular smooth muscle cells (VSMCs) into the intima where they proliferate. The proliferation and migration of VSMCs are fundamental in the extension of atherosclerosis (4, 5).

Shexiang Baoxin pills (SBPs) is a Moschus-based traditional Chinese medicine (TCM) commonly used in the clinical treatment for the relief of cardiovascular diseases (6, 7). SBP includes the following seven medicinal constituents: *Moschus* (*Shexiang*), extract of *Ginseng Radix et Rhizoma(renshen), Bovis Calculus Artifactus* (*Rengong Niuhuang*), *Cinnamomi Cortex* (*Rou Gui*), *Styrax* (*Su Hexiang*), *Bufonis Venenum* (*Chan Su*), and *Borneolum Syntheticum* (*Bing Pian*) (8) (see in Supplementary Table 1). α-Smooth muscle actin (α-SMA) and smooth muscle myosin heavy chain (SM-MHC) are the biomarkers of VSMCs, which suggests the stronger cell differentiation promoted the growth and migration of VSMCs (4, 9, 10). A previous study showed that SBP enhanced the expression of α-SMA and SM-MHC in VSMCs and decrease cell proliferation. Concerning the effects of SBP on VSMC migration, another study demonstrated that SBP could prevent restenosis following stenting by weakening neointimal formation by the migration of VSMCs (11). Although the previous study has illustrated that SBP inhibited the proliferation and migration of VMSCs, the exact effect and the potential pathways of SBP acting on human coronary artery smooth muscle cells (HCASMCs) remain unanswered.

In our study, potential targets and pathways were predicted based on methodologies of network pharmacology and validated by Western blot and molecular docking. Preliminary results demonstrated that 2.5 mg/L SBP acts against ox-LDL-induced proliferation and migration of HCASMCs. Moreover, SBP could function partly through the activation of targets STAT3, MAPK14, and the PI3K/AKT/mTOR pathway. Our current study helps us deepen the understanding of the function of SBP and provide new targets for the treatment of CAD.

## Materials and Methods

### Compositive Compounds of Seven Ingredients in SBP

The chemicals in the seven ingredients in SBP were collected using the Traditional Chinese Medicine Systems Pharmacology Database (TCMSP; http://5th.tcmspw.com/tcmsp.php), Traditional Chinese Medicine Integrative Database (TCMID; http://119.3.41.228:8000/tcmid/), and Shanghai Institute of Organic Chemistry of CAS Chemistry Database [DB/OL] (http://www.organchem.csdb.cn). We collected the Canonical SMILES format of all chemicals in PubChem (http://pubchem.ncbi.nlm.nih.gov) to obtain the corresponding molecular structures. More importantly, we screened the chemicals in SwissADME (http://www.swissadme.ch/index.php) according to pharmacokinetics and drug likeness. Chemicals can be used for further research only if they meet the following criteria: gastrointestinal absorption is high, and at least two of Lipinski, Ghose, Veber, Egan, and Muegge should be assessed as “yes.”

### Drug and CAD Targeting Genes

Since we collected the potential chemicals of SBP, inputting their Canonical SMILES format into the SwissTargetPrediction website (http://www.swisstargetprediction.ch) is a way of getting the targets of each chemical. Targets whose probability is above 10% would be selected as potential targets.

To acquire the CAD target genes, we searched with the keyword “coronary artery disease” from five disease databases containing GeneCards (https://www.genecards.org), OMIM (https://omim.org), TTD (http://db.idrblab.net/ttd/), PharmGkb (https://www.pharmgkb.org), and Drugbank (https://go.drugbank.com). The target genes obtained from five databases were then merged into CAD targets datasets. Finally, we picked these genes presenting in both the SBP target and CAD target datasets as the targets wherein SBP exhibited the therapeutic effects on CAD.

### Compounds–Targets Network Construction

The compounds–targets network between compounds and targets was visualized using Cytoscape (version 3.7.4). In the network, the nodes symbolize compounds and targets, and the interactions between compounds and targets are represented by the edges.

### Protein–Protein Interaction Network Construction

The potential targets of SBP on CAD were inserted into the STRING website (http://string-db.org/). The information concerning protein interaction and score of interaction was input into Cytoscape to construct the PPI network, and the detection of the topology of the network was conducted. Moreover, the proteins imported into Cytoscape were subsequently screened by the standard that the index of each protein, including betweenness centralities (BC), closeness centralities (CC), degree centralities (DC), eigenvector centralities (EC), and local average connectivity-based method centralities (LAC), is above the median. We finally revealed the primary target proteins with twice selection using the same screen criteria in R (version 4.0.2).

### Pathway Enrichment Analyses

The Kyoto Encyclopedia of Genes and Genomes (KEGG) (https://www.kegg.jp/kegg/) was the tool used for pathway enrichment analysis. Pathways with *q* < 0.05 were screened. Gene Ontology (GO) analysis was performed with clusterprofiler package, and the top 20 pathways and top 20 GO terms were visualized with R (Version 4.0.2).

### Molecular Docking

Molecular docking was performed with the AutoDockTools-1.5.6 following the principle of semiflexibility. Another software, PyMOL (version 2.4.0), was used for predocking component proteins and small molecules. The PPI network was applied to select the candidate targets. The candidate targets were then inserted into the RSCB PDB (https://www.omicshare.com) website to obtain the PDB format of the protein, making the molecular docking prediction more reliable. The components' 3D structures were obtained from the PubChem database (https://pubchem.ncbi.nlm.nih.gov/) and processed with a software called ChemDraw. The optimized structures were calculated using AutoDockTools-1.5.6 by hydrogenating, charging, and calculating the rotatable bond number. Subsequently, the removal of non-protein molecules and ligands in the genes was conducted with PyMOL (version 2.4.0), and AutoDockTools-1.5.6 was applied to detect the total charge and add hydrogen. With the clustering tool of AutoDockTools-1.5.6, the lowest energy pose was selected as the candidate molecular structure. Through PyMOL, we obtained the optimized two-dimensional (2D) and 3D structures of proteins and molecules to reveal the corresponding binding bonds and protein residues. Binding energy tends to be more stable once their binding free energy of docking is <-4 Kcal/mol.

### SBP Preparation

SBP powder was provided by Shanghai Hutchinson Pharmaceuticals Co. Ltd. (Shanghai, China; manufacturer batch number: 200722; NMPA approval number Z31020068). One milligram of SBP powder was weighed and dissolved into 2 ml of dimethyl sulfoxide (DMSO) to obtain 0.5 mg/ml SBP solution. The SBP solution (0.5 mg/ml) was diluted to the working concentration with DMEM (Gibco, USA) (DMSO contents were <0.1%). The 0.22-μm filter (Millipore, USA) was used for filtration and sterilization of the working solution.

### HCASMC Culture and Stimulation

HCASMCs were obtained from Sigma. We cultured the HCASMCs with a humidified incubator at 37°C provided with 5% CO_2_ growing inappropriate volume of DMEM (Gibco, USA) containing 10% fetal bovine serum (FBS, Gibco) and 1% streptomycin (100 μg/ml) and penicillin (100 U/ml) (Beyotime, Shanghai, China) bought from Yiyuan Biotechnologies (Guangzhou, China) to create an unusual lipid environment and contribute to proliferation and migration models. The SBP-treated group was cultured with extra 0, 1, 2.5, 5, and 7.5 mg/L SBP for 0, 12, 24, 36, and 48 h to explore the effect of SBP on the proliferation and migration of HCASMCs.

### Cell Counting Kit-8 Analysis

To test the cell viability and proliferation, CCK-8 was carried out with a CCK-8 assay kit (APExBIO, USA). HCASMCs in 100 μl were cultured in a 96-well microplate (5,000 cells/well) (Corning, NY, USA). The absorbance at 450 nm every 12 h was detected by a microplate reader (Multiskan FC, Thermo Fisher Scientific) under the manufacturer's direction.

### Cell Cycle Analysis

Three groups of cells were cultured with DMEM basic medium, ox-LDL (50 mg/L), and SBP (2.5 mg/L), respectively, for 24 h. HCASMCs (1 × 10^6^) were collected using 0.25% trypsin–EDTA (Beyotime, Shanghai, China) from the six-well plates (Corning, NY, USA). Detached cells were clustered and washed with PBS (Beyotime, Shanghai, China) and then centrifuged twice (2,000 rpm, 5 min). The HCASMCs were fixed in 70% ethanol overnight at 4°C. The fixed HCASMCs were centrifuged for 3 min at 1,000 rpm and resuspended with PBS for removal of residual ethanol. The centrifuged cells were then incubated with 500 μl of working fluid including 10% RNase A and 90% propidium iodide (KeyGen BioTECH, Nanjing, China) for 1 h in the dark. The cell cycle analysis was processed with a flow cytometer (Gallios Flow Cytometer; Beckman Coulter, USA), and data were analyzed with the suggested software (Kaluza for Gallios; Beckman Coulter, USA).

### Transwell Assay

HCASMCs were cultured in six-well plates and treated with 50 mg/L ox-LDL for 24 h. The group treated with 2.5 mg/L SBP and 50 mg/L ox-LDL grew in six-well plates for 24 h. Then 200 μl of cells (1 × 10^5^ cells/ml) was seeded into the upper compartment of Transwell chambers (3,422, Corning, New York, USA). DMEM FBS-free (500 μl) was added to the lower chambers, and the wells were incubated at 37°C with 5% CO_2_ for 24 h. After removal of the medium, the HCASMCs above the filter membrane were removed with cotton swabs, and the HCASMCs that migrated to the lower side was fixed within 4% glutaraldehyde for 40 min. Finally, the cells were stain fixed in 0.1% crystal violet for 35 min and the extra crystal violet was removed with PBS, and then the cells adherent to the lower side were calculated and photographed with an Olympus-CKX53 microscope.

### Western Blot

HCASMC cells were distributed in six-well plates at 5 × 10^5^/dish, and the corresponding treatment was carried out for 24 h. Then, cell lysis was conducted with RIPA lysis kit (Beyotime) including phenylmethanesulfonyl fluoride. Quantification of total protein was detected with BCA protein assay kit (Beyotime). Total proteins (20 μg) were separated using 7.5–10% sodium dodecyl sulfate polyacrylamide gel electrophoresis. The resolved protein was subsequently transferred onto a nitrocellulose membrane. Membranes were blocked with quick blocking buffer (Beyotime) for 10 min at room temperature, and then incubated with primary antibodies at 4°C for 15 h, including p-STAT3, p-MAPK14, p-AKT (1:1,000; Cell Signaling Technology, Danvers, MA, USA), p-PI3K, PI3K, AKT, mTOR, and p-mTOR (1:1,000; Proteintech Group, Wuhan, China). Membranes were washed and then incubated in secondary antibodies (1:10,000) at room temperature for 1 h. Proteins were imaged by the Bio-Rad Chemi Doc XRS imaging system (Bio-Rad, USA).

### Statistical Analysis

Statistical analyses were processed with SPSS (version 16.0), GraphPad Prism (version 8.0), and R software (version 4.0.2). Data normality and homogeneity of variance were explored with the Shapiro–Wilk W-test. Statistical analyses were performed with *t*-test (parametric unpaired or paired, two groups of analysis) and Mann–Whitney *U*-test (non-parametric unpaired). Multiple-group analysis was performed with ANOVA. When a *p*-value was lower than 0.05, the result was considered statistically significant.

## Results

### Filtering of Active Constituents of SBP on CAD

**Figure 1:**
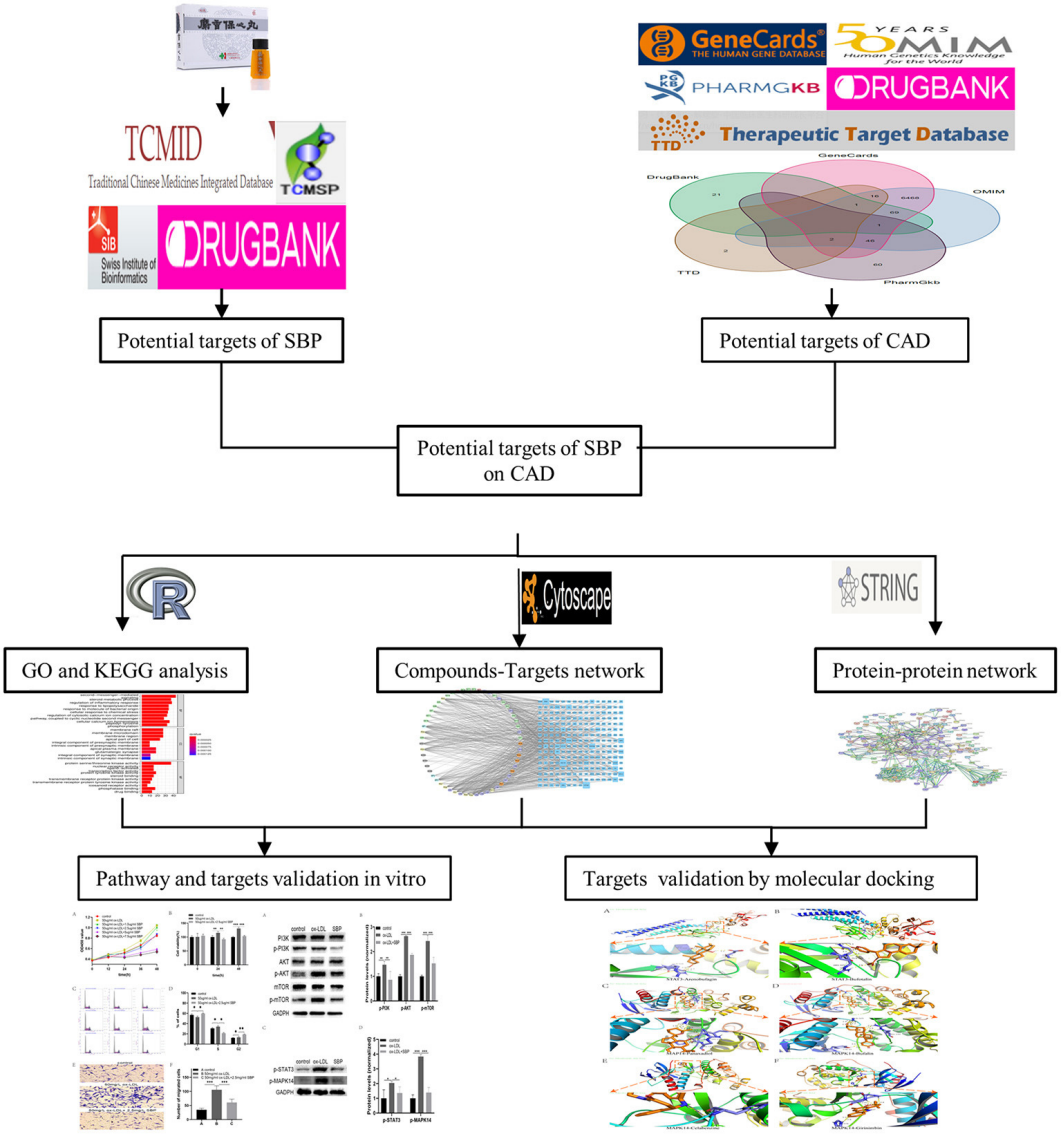
Flowchart of the network pharmacology-based study.

**Figure 2:**
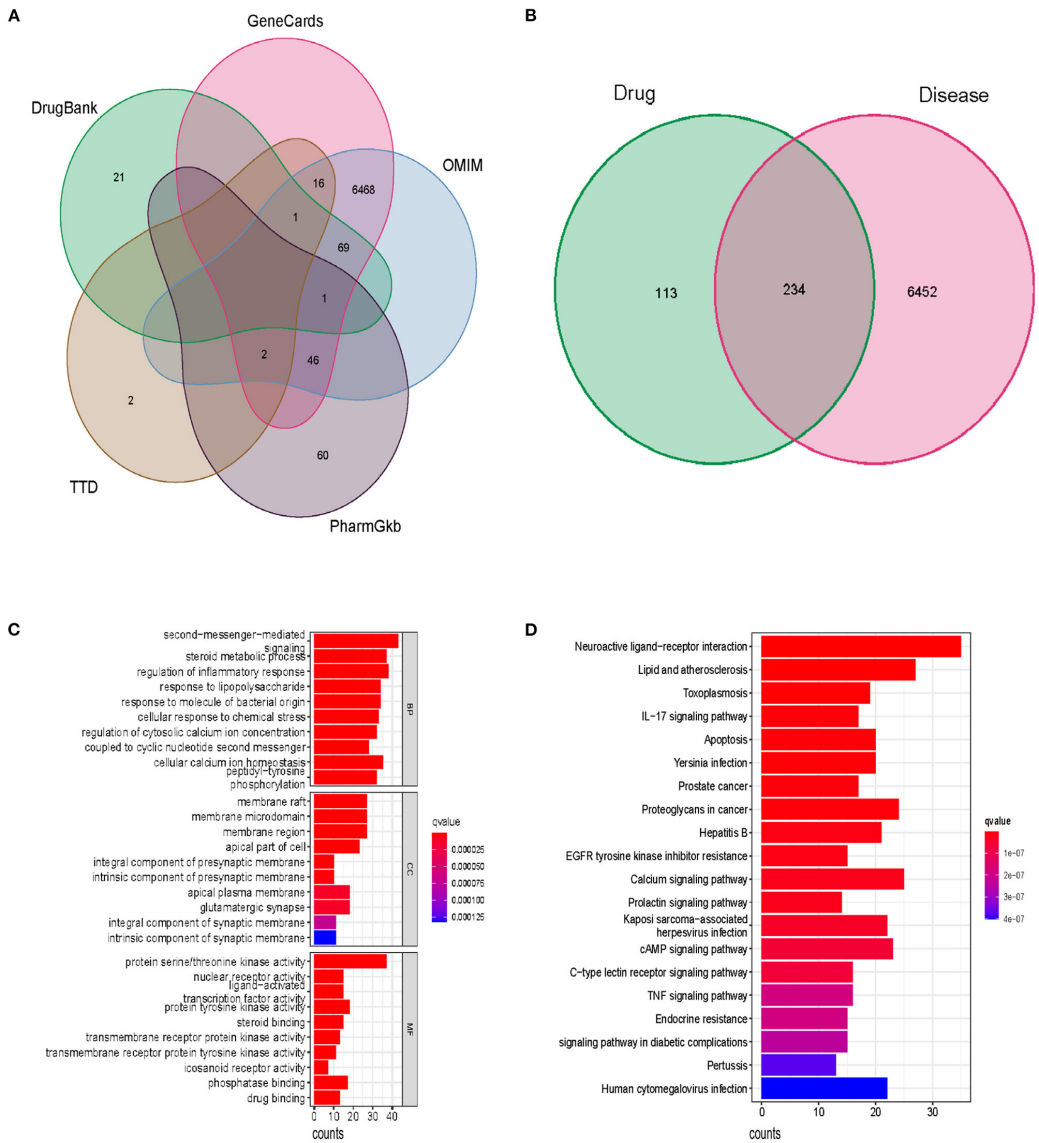
The potential function and pathways of SBP on CAD. **(A)** Disease genes of CAD from five databases. **(B)** Genes existing in both drug and disease. **(C)** Top 10 results of the GO analysis including BP, GC, and MF. **(D)** Top 30 pathways in which SBP functions in CAD.

The flow chart of our study has been displayed in Figure 1. Following the ADME thresholds of drug likeness (DL) ≥ 0.18 and oral bioavailability (OB) ≥ 30%, 106 compounds of SBP were collected from the three widely used databases: TCMSP, TCM Database@Taiwan, and TCMID after excluding the unsuitable compounds, as displayed in Supplementary Table 2. A total of 6,686 CAD targets from five databases including GeneCards, OMIM, PharmGkb, TTD, and Drugbaresunk were collected and merged into the CAD disease dataset (Figure 2A). Common targets of both CAD and the chemical constituents were considered potential targets of SBP on CAD. Genes existing in both SBP and CAD were 234 in all (Figure 2B).

### GO and KEGG Enrichment Analysis

GO enrichment analysis identified functional targets involved in cellular composition (CC), molecular function (MF), and biological process (BP). Among the BP section, the second messenger-mediated signaling, steroid metabolic process, and regulation of inflammatory response are the most significant functions of SBP, suggesting that SBP may relieve the inflammation in the coronary, which contributes to the development of CAD. Results of the MF analysis displayed that protein serine/threonine kinase activity is the primary function pattern of SBP in the treatment of CAD (Figure 2C). In terms of KEGG analysis, the apoptosis pathway and the PI3K/AKT/mTOR pathway were found to be related significantly to the treatment of CAD with SBP (Figure 2D and Supplementary Table 3).

### Ingredient–Target and PPI Network

**Figure 3:**
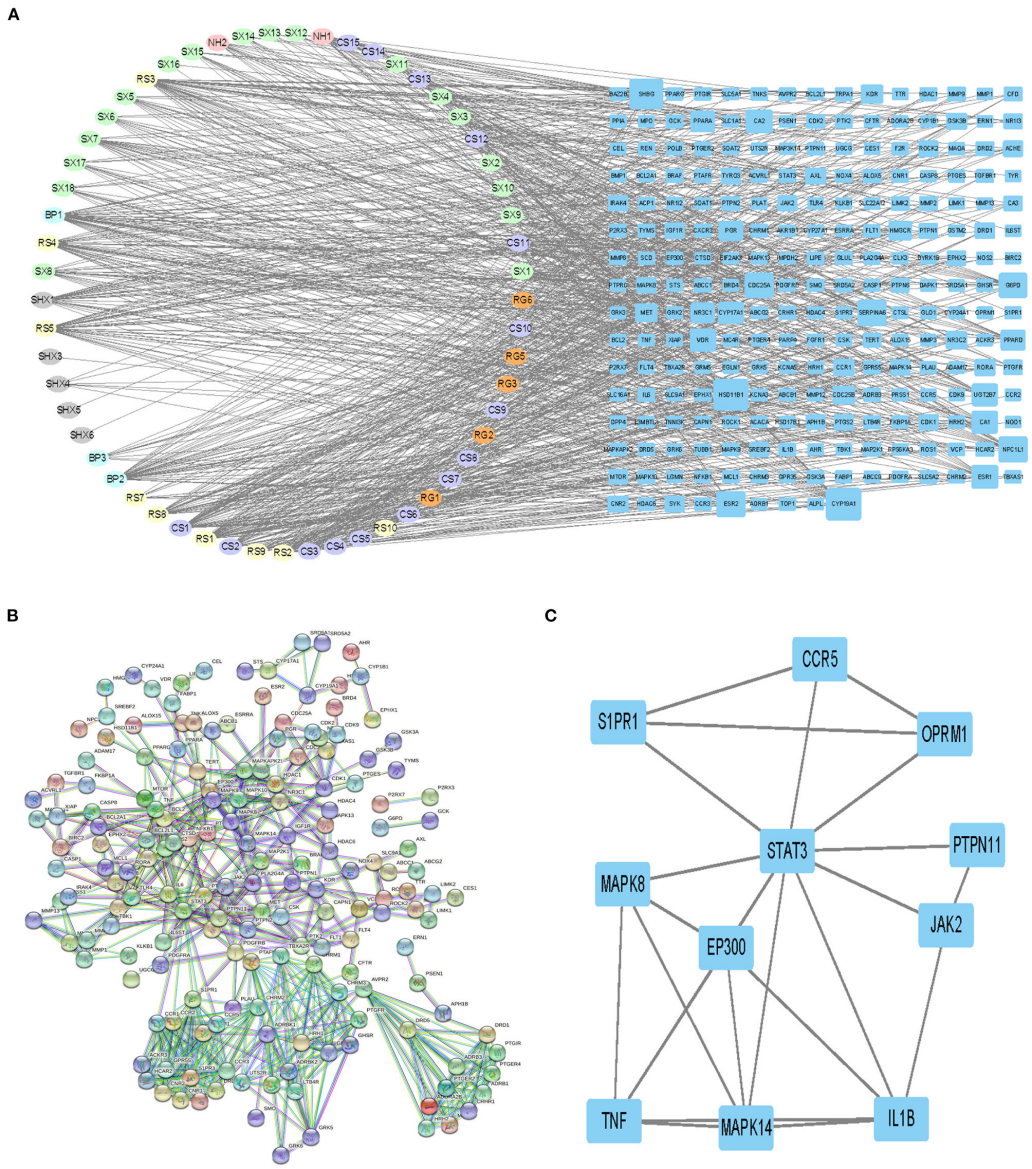
Ingredients–targets network and selecting of primary effective targets by PPI. **(A)** Network revealing the interaction between ingredients and potential targets. The nodes with different colors in oval shapes represent the different ingredients of SBP. Nodes with blue color in rectangle shapes are the potential targets of SBP on CAD, and the area of each node is positive with their degree. Edges were used to indicate the correlation between different nodes. **(B)** PPI network diagrams produced by STRING. **(C)** Network of primary targets of SBP functioning on CAD.

Figure 3A demonstrates that among 106 compounds of SBP, 57 compounds play the potential role for the treatment of SBP (Figure 3A), and the corresponding relationship between nodes in oval shapes is displayed in Table 1. As shown in the network, HSD11B1, CYP19A1, and SHBG are the targets that most ingredients are predicted to act on. In terms of Figure 3B, 234 target genes related to SBP on CAD were uploaded to STRING to produce a PPI network. The PPI network contains 613 interactions, and each interaction shares a combined score >0.9. Subsequently, the PPI network generated by STRING was imported into Cytoscape (version 3.7.2) for further analysis. Finally, 11 genes were preserved and considered as the key gene after twice selection with screening criteria that BC, CC, DC, EC, and LAC of each selected gene are above the median (Figure 3C, Supplementary Figures 1, 2), and the gene name is presented in Table 2.

**Table 1:** Chemical names of symbols and their sorts.

**Sorts**	**Symbol**	**Chemical name**
Bingpian	BP1	Asiatic acid
	BP2	Dipterocarpol
	BP3	Bronyl acetate
Chansu	CS1	Cinobufotalin
	CS2	Cinobufagin
	CS3	Cinobufaginol
	CS4	Q-bufarenogin
	CS5	Arenobufagin
	CS6	Epinephrine
	CS7	Resibufogenin
	CS8	5-hydroxytryptamine
	CS9	7α-hydroxycholesterol
	CS10	7β-hydroxycholesterol
	CS11	Helleprigenin
	CS12	Bufotalin
	CS13	Bufalin
	CS14	Bufotenine
	CS15	Bufotenidine
Niuhuang	NH1	Deoxycholic acid
	NH2	Cholic acid
Renshen	RS1	Gomisin B
	RS2	Suchilactone
	RS3	Kaempferol
	RS4	Celabenzine
	RS5	Deoxyharringtonine
	RS7	Frutinone A
	RS8	Girinimbin
	RS9	Panaxadiol
	RS10	Fumarine
Rougui	RG1	Syringaresinol
	RG2	Coumarin
	RG3	Cinnamic acid
	RG5	Cinnamaldehyde
	RG6	Protocatechuic acid
Shexiang	SX1	Methyl palmitate
	SX2	Cholic acid
	SX3	3α-hydroxyandrostan-4-en-17β-one
	SX4	3α-hydroxy-5α-androstan-17-one
	SX5	3β- hydroxy-5β-androstan-17-one
	SX6	3α- hydroxy-5β-androstan-17-one
	SX7	3β- hydroxy-androst-5-en-17-one
	SX8	
	SX9	Aspartate
	SX10	Normuscone
	SX11	androst-4-en-3,17-dione
	SX12	5α-androstane-3,17-dione
	SX13	5β-androstane-3,17-dione
	SX14	5β-androstane-3α,17β-diol
	SX15	5α-androstane-3β,17α-diol
	SX16	Muscone
	SX17	5α-androstane-3,17-diol
	SX18	5β-androstane-3α,17α-diol
Suhexiang	SHX1	Styracin
	SHX3	Cinnamic acid
	SHX4	Methyl cinnamate
	SHX5	Cinnamaldehyde
	SHX6	Ethylphenol

**Table 2:** Candidate targets of SBP on CAD screened by Cytoscape and their degrees.

**Protein name**	**Gene name**	**Degree**
Signal transducer and activator of transcription 3	STAT3	10
Mitogen-activated protein kinase 14	MAPK14	5
Interleukin-1 beta	IL1B	5
EP300-interacting inhibitor of differentiation 1	EP300	5
Tumor necrosis factor	TNF	5
Mitogen-activated protein kinase 8	MAPK8	4
Tyrosine-protein kinase JAK2	JAK2	3
C-C chemokine receptor type 5	CCR5	3
Mu-type opioid receptor	OPRM1	3
Sphingosine 1-phosphate receptor 1	S1PR1	3

### The Proliferation of HCASMCs Stimulated by ox-LDL Was Inhibited by SBP

**Figure 4:**
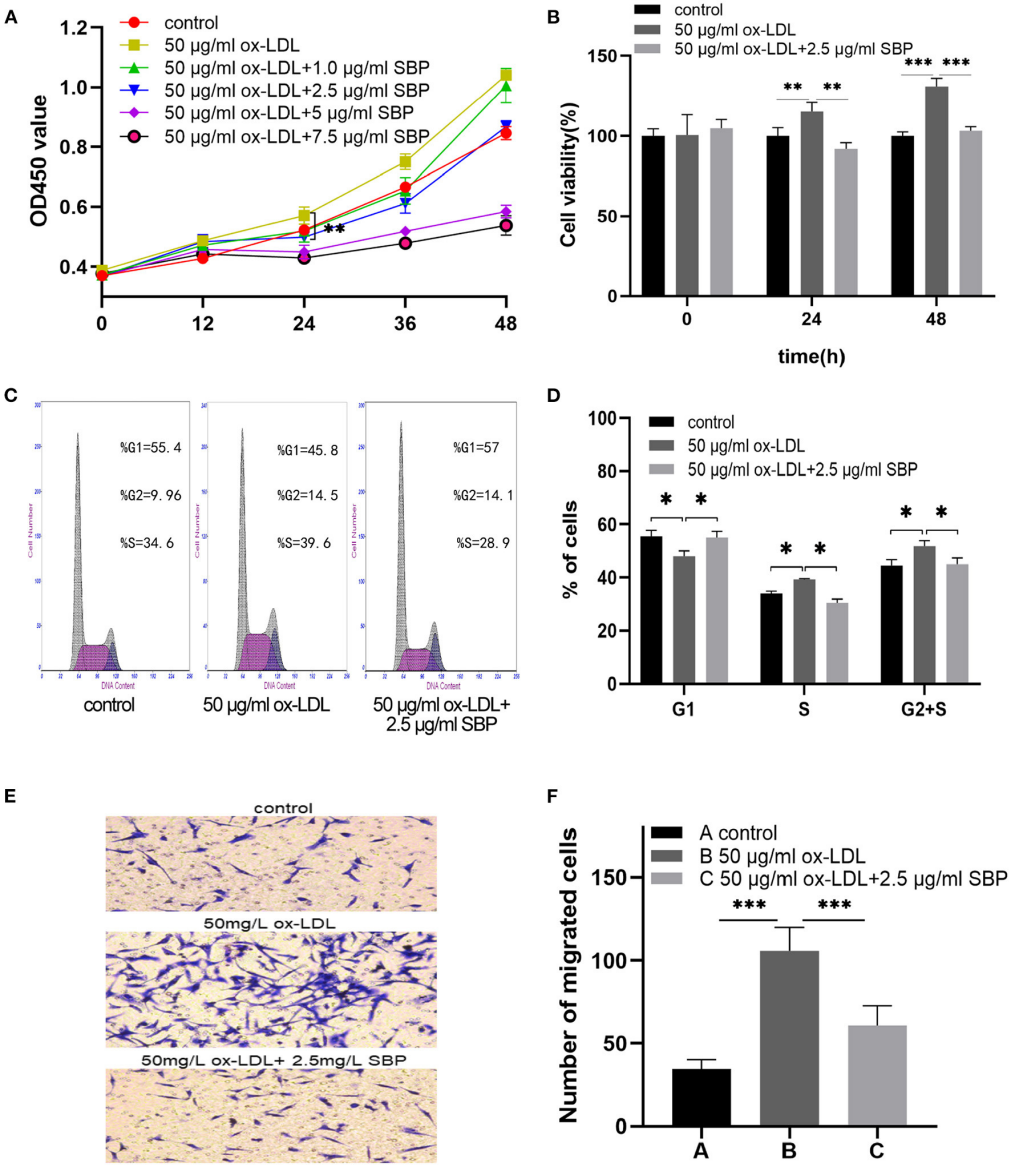
SBP suppressed the proliferation and migration stimulated by ox-LDL. **(A)** Cell proliferation was tested using CCK-8 cell proliferation assays. The proliferation of HCASMC was activated by ox-LDL, and SBP reversed the effect of ox-LDL in cell multiplication. **(B)** The cell viability among groups at 0, 24, and 48 h was exhibited in histogram B. **(C)** Cell cycle assay is presented. **(D)** Plotted cell cycle statistical analysis, suggesting that SBP relieved the stimulation of ox-LDL in HCASMCs. **(E)** Cells that migrated to the lower side of the filter membrane. **(F)** Number of migrated cells in the Transwell assay is calculated. *p < 0.05, **p < 0.01, ***p < 0.001.

As shown in Figures 4A,B, the growth curves present that ox-LDL promoted the proliferation of the HCASMCs with a concentration of 50 mg/L for 24 h, and SBP shared the significant reduction in the acceleration of ox-LDL with a concentration of 2.5 mg/L.

Besides, the results, as shown in Figures 4C,D, indicated that ox-LDL accelerated the cell cycle from the G_1_ phase to the S and G_2_ phases. Meanwhile, HCASMCs treated with 2.5 mg/L SBP and 50 mg/L ox-LDL were arrested in the G_1_ phase.

### SBP Suppressed the Migration of HCASMCs Induced by ox-LDL

As is evidenced in Figures 4E,F, after pretreating with 50 mg/L ox-LDL for 24 h, HCASMCs treated with ox-LDL migrated more easily from the upper chamber to the lower chamber compared with the control. In the contrast, HCASMCs preconditioned with 2.5 mg/L SBP and 50 mg/L ox-LDL inhibited transmigration of HCASMCs from the upper chamber to the lower chamber compared with cells preconditioned with 50 mg/L ox-LDL.

### Western Blot Validation

**Figure 5:**
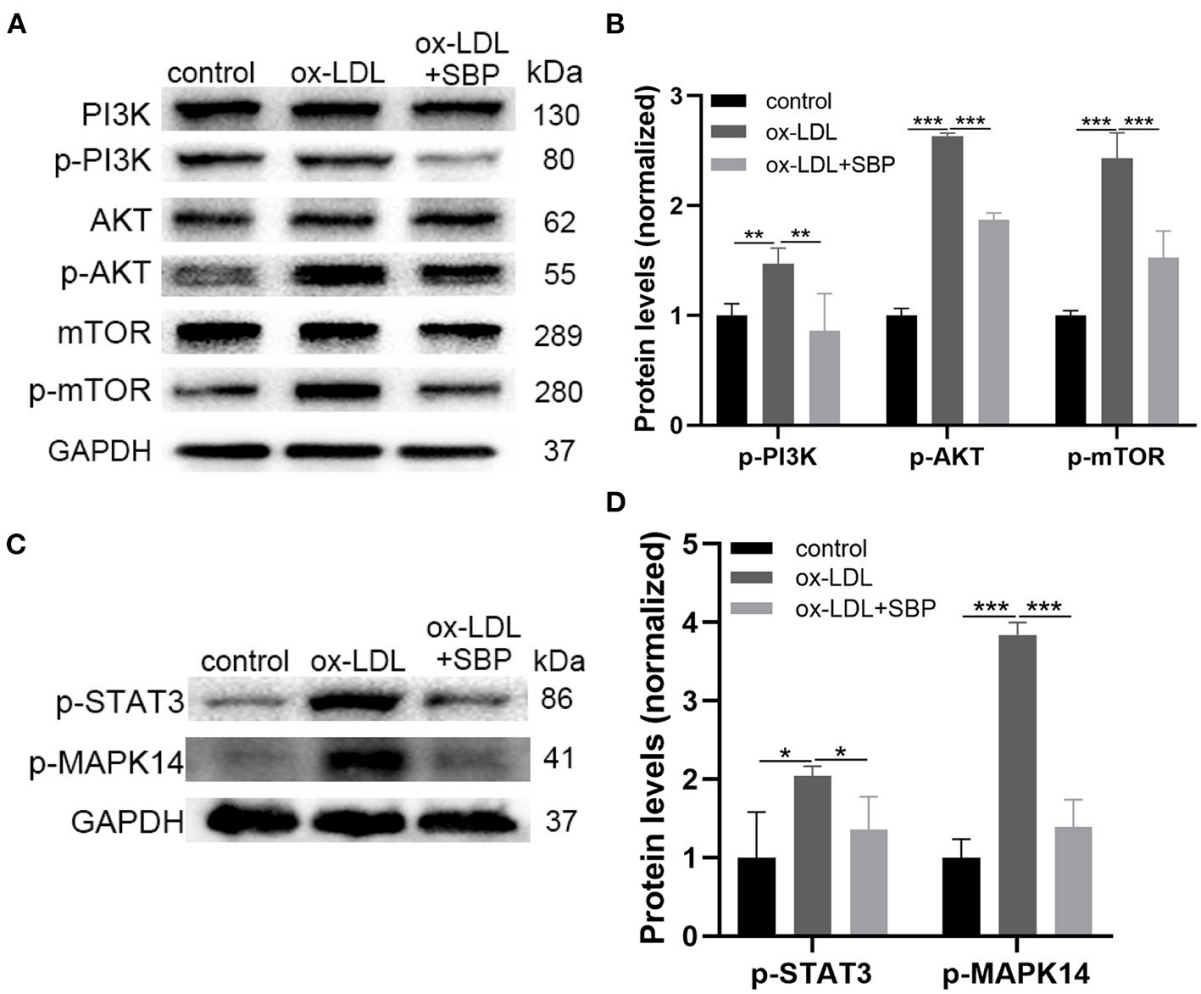
The prediction targets and pathways of network pharmacology were validated by Western blot. **(A)** The expression of PI3K, p-PI3K, AKT, and p-AKT, mTOR, p-mTOR was detected by Western blot. **(B)** The significant difference of p-PI3K, p-AKT, and p-mTOR was revealed among the three groups. **(C)** Expression of top 2 targets including STAT3 and MAPK14 was investigated by Western blot. **(D)** Statistical analysis of Western blot. *p < 0.05, **p < 0.01, ***p < 0.001.

Figures 5A,B demonstrated that protein expression of unphosphorylated PI3K/AKT/mTOR did not change significantly among the three groups, while the expression of activated PI3K/AKT/mTOR with phosphorylation in ox-LDL-induced HCASMCs increased significantly than the control. Moreover, the addition of SBP suppressed the activation of the PI3K/AKT/mTOR pathway in ox-LDL-stimulated HCASMCs. These results suggested that ox-LDL increased the proliferation of HCASMCs through activating the PI3K/AKT/mTOR pathway, and SBP inhibited the phosphorylation of the pathway to suppress the multiplication of the cells.

Figures 5C,D presents the protein expression of phosphorylated STAT3 and MAPK14. A significant increase was found between the ox-LDL group and the control group. In addition, SBP inhibited the phosphorylation of STAT3 and MAPK14, indicating SBP targeted the two proteins to suppress the growth of HCASMC induced by ox-LDL.

### Molecular Docking

**Figure 6:**
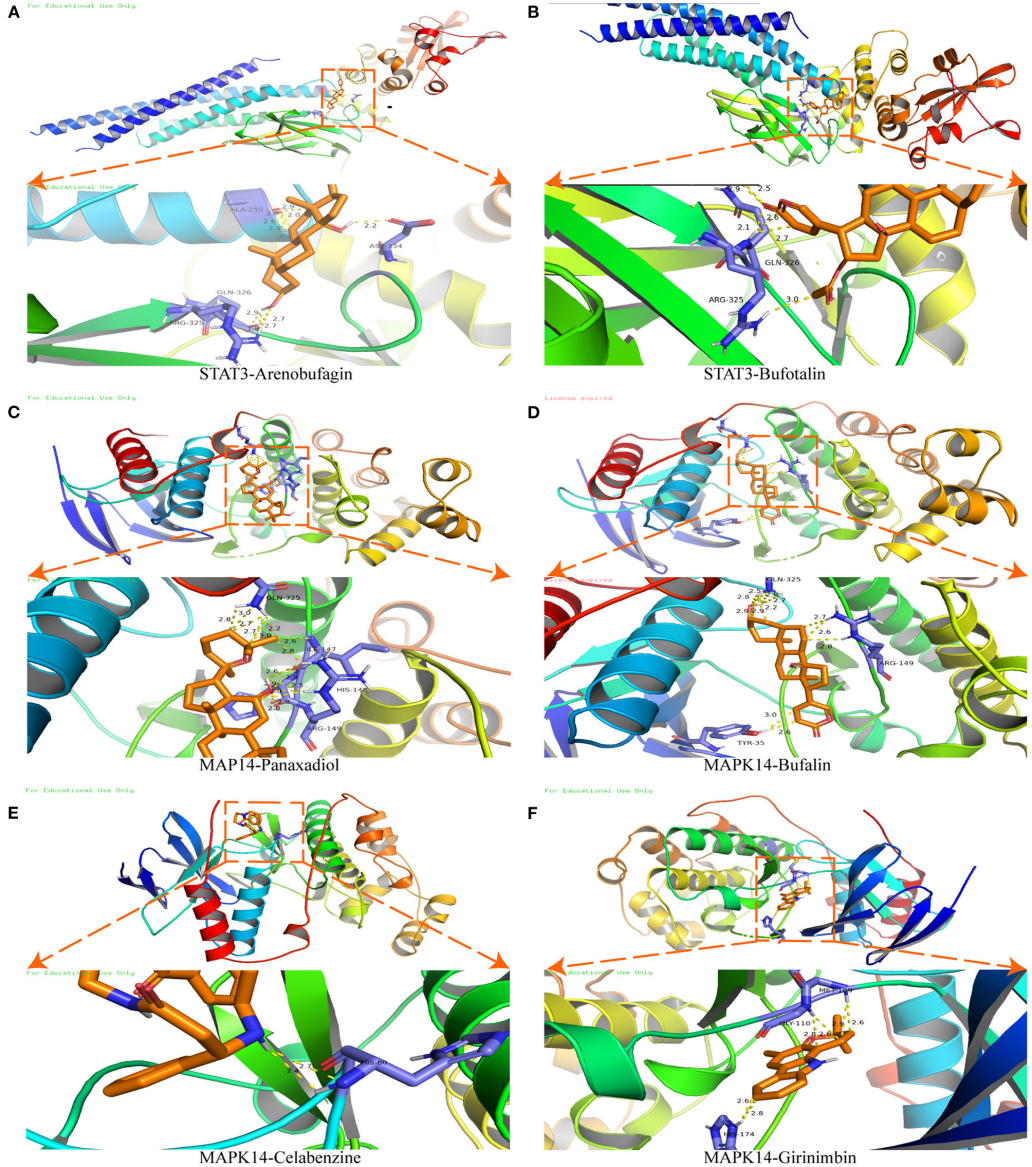
Molecular docking of STAT3 and MAPK14. **(A)** Molecular docking between STAT3 and arenobufagin. **(B)** Molecular docking between STAT3 and bufotalin. **(C)** Molecular docking between MAPK14 and panaxadiol. **(D)** Molecular docking between MAPK14 and bufalin. **(E)** Molecular docking between MAPK14 and celabenzine. **(F)** Molecular docking between MAPK14 and MAPK14-girinimbin.

Molecular docking was used to verify the binding action mode of STAT3 and MAPK14 with their molecular candidates, respectively, dependent on the network analysis. The findings demonstrated that STAT3 interacted with arenobufagin and bufotalin, as shown in Figures 6A,B, respectively. As displayed in Figure 6A, the structure of arenobufagin could form bonds with ALA-250, ASP-334, GLN-326, and ARG-325 in STAT3. In Figure 6B, the structure of bufotalin could generate bonds with ARG-325 and GLN-326 in STAT3. As illustrated in Figures 6C–F, bonds were formed between MAPK14 and panaxadiol, bufalin, celabenzine, and girinimbin. As displayed in Figure 6C, the structure of panaxadiol could form bonds with GLN-325, ARG-149, and HIS-148 in MAPK14, respectively. Also, in Figure 6D, bonds were generated to bind bufalin with TYR-35, ARG0-149, and GlN-325 in MAPK14. Moreover, celabenzine interacted with HIS-80 in MAPK14 (Figure 6E). As we can see in Figure 6F, girinimbin changes the structure of MAPK14 by interacting with MET-109, GLY-110, and HIS174. In conclusion, an interplay exists between ingredients and candidate targets through different bonds. The binding energy was calculated to estimate the matching degree of proteins with ingredients. The detailed binding energy score is presented in Table 3. Lower binding energy suggested greater stability. The results validated that the ingredients could bind to the active site of potential targets.

**Table 3:** The binding energy between proteins and chemical molecules in molecular docking.

**Protein**	**Chemical name**	**Binding energy (kcal/mol)**
STAT3	Arenobufagin	−8.3
STAT3	Bufotalin	−7.8
MAPK14	Panaxadiol	−7.7
MAPK14	Bufalin	−7.6
MAPK14	Celabenzine	−7.2
MAPK14	Girinimbin	−9.6

## Discussion

To clarify the effect of SBP on HCASMCs, we functioned ox-LDL as stimulation of HCASMCs and attempted to inhibit the proliferation and the migration induced by ox-LDL on HCASMCs with SBP. The current study found that 50 mg/L ox-LDL succeeded in stimulating the migration and proliferation of the HCASMCs. Meanwhile, SBP prevented the ox-LDL-induced migration and proliferation of the HCASMCs. Network pharmacology predictions have shown that 11 proteins could be the major targets of SBP on CAD. To prove the accuracy of the network pharmacology predictions, the top 2 targets including Stat3 and MAPK14 have been validated by experiments *in vitro*. Results of the Western bolt assay revealed that p-Stat3 and p-MAPK14 were activated by ox-LDL and could be partly suppressed by ox-LDL. Moreover, the PI3K/AKT/mTOR pathway was calculated as the potential functional pathway in the treatment of CAD by SBP. The phosphorylated protein expression of PI3K/AKT/mTOR in ox-LDL was significantly higher than the control group and the SBP group, suggesting that the mechanism of ox-LDL contributing to the proliferation of HCASMCs was through activating the phosphorylation of the PI3K/AKT/mTOR pathway, and the inhibition of the PI3K/AKT/mTOR pathway may be the reason SBP suppresses the proliferation of HCASMCs induced by ox-LDL.

In our research, the CCK-8 assay was applied for the measurement of cell proliferation, and 2.5 mg/L SBP can significantly inhibit the proliferation of HCASMSCs stimulated by ox-LDL. Meanwhile, HCASMSCs suppressed by 2.5 mg/L SBP presented no statistical difference with the control group, which avoided the toxicity that resulted from the high concentration of SBP. Moreover, 2.5 mg/L SBP could contribute to the significant difference in protein expression among the three groups. This also accords with earlier observations, which showed that α-SMA and SM-MHC were overexpressed in VSMCs after treating VSMCs with several concentrations of SBP (12). Similarly, another report also showed that SBP upregulated the expression of α-SMA and SM-MHC while inhibited the cell cycle at the G_1_ phase, which indicated the suppression of VSMCs (13). The current study also found that SBP inhibited the migration of HCASMCs stimulated by ox-LDL. On the contrary, a previous study showed that SBP promotes proliferation, migration, and adhesion than the control of endothelial progenitor cells. A possible explanation for this might be that SBP exerts different functions among various cell lines.

STAT3 is a member of the STAT family, which is fundamentally active among cancers such as breast, lung, gastric, and prostate cancers (14–16). Despite the extensive function of STAT3 among several cancer cells, growing evidence has disclosed the contribution of activated STAT3 to cancer cell proliferation and aberrantly activated STAT3 is related to tumor malignancy (17–19). Except for tumors, STAT3 also plays an important role in the proliferation of SMCs. Suppression of STAT3 signaling was reported to prevent the proliferation of VSMCs (20), which followed our results that SBP inhibited the proliferation of HCASMCs with the dysregulation of phosphorylated STAT3.

MAPK14 is also known as p38 mitogen-activated protein kinase (p38MAPK), even though several studies have revealed that the loss of P38MAPK resulted in the proliferation of cell lines including osteoblasts and erythroid cells (21). On the contrary, researches of MAPK14 related to SMCs showed that VSMC differentiation and proliferation were inhibited both *in vivo* and *in vitro* (22), which is in accordance with human leiomyoma cells (23). Similar to the recent report, in our study, the expression of phosphorylated MAPK14 presented upregulated in ox-LDL-induced HCASMCs, contributing to cell proliferation, and downregulated when treated with SBP, resulting in cell suppression.

The PI3K/AKT/mTOR pathway regulated the multifaceted functions of cells like cell cycle, cellular proliferation, growth (24). A recent study illustrated that increasing the phosphorylation of this pathway promotes the growth of breast cancer cells (25). Also, researches revealed that the activation of the PI3K/AKT/mTOR pathway promotes the multiplication among SMCs (26), which is verified in our study (27).

Although the present study shed some light on the protective effect of SBP on CAD, it has some drawbacks. First of all, the predicted targets and pathways were only verified in HCASMCs, and further verification is needed to be conducted *in vivo*. Moreover, the functional targets and pathways presented in this study are limited to HCASMCs. It is needed to be verified experimentally whether they share the same effect on other cell lines associated with CAD.

In conclusion, methodologies of network pharmacology were performed to predict the potential targets and pathways, detecting 11 primarily effective proteins and pathways functioning in the treatment of SBP on CAD. We chose the top 2 targets and one pathway to perform experimental verification. Besides, SBP inhibited the ox-LDL-induced proliferation and migration of HCASMCs, which was evidenced by the CKK-8 assay, cell cycle, and Transwell assay. The weakened proliferation and migration of HCASMCs could relieve the symptoms of CAD coronary stenosis, which might be one of the mechanisms of SBP on CAD. The target proteins and pathways of SBP provide a new direction for the treatment of CAD.

## Conclusion

In this study, we predicted and verified that targets STAT3, MAPK14, and the PI3K/AKT/mTOR pathway are the effective therapeutic targets of SBP on CAD. Pathways network analysis illustrated that the PI3K/AKT/mTOR pathway is related to the treatment of SBP, which is also detected in HCASMCs. The *in vitro* experiment verified that SBP inhibited the HCASMCs from proliferation and migration induced by ox-LDL. The present study deepens the understanding of the mechanisms of SBP on CAD. Moreover, the role of HCASMCs should be valued during CAD, and the potential treatment target of CAD has been provided in this research.

## Data Availability Statement

The original contributions presented in the study are included in the article/Supplementary Material, further inquiries can be directed to the corresponding author/s.

## Author Contributions

EJ conceived the study. LH and YZ initially drafted the manuscript and performed the experimental assay. CH, JC, YW, and SZ analyze data under the supervision of HZ and SH. All authors contributed to the article and approved the submitted version.

## Conflict of Interest

The authors declare that the research was conducted in the absence of any commercial or financial relationships that could be construed as a potential conflict of interest.

## Publisher's Note

All claims expressed in this article are solely those of the authors and do not necessarily represent those of their affiliated organizations, or those of the publisher, the editors and the reviewers. Any product that may be evaluated in this article, or claim that may be made by its manufacturer, is not guaranteed or endorsed by the publisher.
