# Supplementary material for: Shexiang Baoxin Pills Inhibited Proliferation and Migration of Human Coronary Artery Smooth Muscle Cells via PI3K/AKT/mTOR Pathway
Source: Front Cardiovasc Med. 2021 Aug 25;8:700630. doi: 10.3389/fcvm.2021.700630 (PMC8425485; doi:10.3389/fcvm.2021.700630)
Supplement: Supplementary file 1 [file Table_1.docx]

**Table S1** Components of the Shexiang Baoxin pill

| ENG name (Pharmacopeia of China 2015) | CHN Pingyin  name | Latin name of original plant or animal (Validated MPNS name) | Family name | Medicinal part |
| --- | --- | --- | --- | --- |
| *Moschus* | She Xiang | *Moschus berezovskii* Flerov, *Moschus sifanicus* Przewalski or *Moschus moschiferus* Linnaeus | Cervidae | Secretion |
| *Bovis Calculus Artifactus* | Rengong Niuhuang | N/A | N/A | N/A |
| *Styrax* | Su Hexiang | *Liquidambar orientalis* Mill. | Hamamelidaceae | Resin |
| *Ginseng Radix et Rhizoma* | Ren Shen | *Panax ginseng* C.A.Mey. | Araliaceae | Radix & Rhizome |
| *Cinnamomi Cortex* | Rou Gui | *Cinnamomum cassia* (L.) J.Presl | Lauraceae | Bark |
| *Bufonis Venenum* | Chan Su | *Bufo bufo gargarizans* Cantor or *Bufo melanostitus* Schneider | Bufonidae | Secretion |
| *Borneolum Syntheticum* | Bing Pian | N/A | N/A | N/A |

N/A : Not applicable.
