# Supplementary material for: Shexiang Baoxin Pills Inhibited Proliferation and Migration of Human Coronary Artery Smooth Muscle Cells via PI3K/AKT/mTOR Pathway
Source: Front Cardiovasc Med. 2021 Aug 25;8:700630. doi: 10.3389/fcvm.2021.700630 (PMC8425485; doi:10.3389/fcvm.2021.700630)
Supplement: Supplementary file 2 [file Table_2.docx]

Table S2 106 chemical components from 7 sorts in SBP.

| Sorts | Chenmical name | | | |
| --- | --- | --- | --- | --- |
| Shexiang | methyl palmitate | aspartate | glycine | serine |
|  | glutamic acid | urea | normuscone | cholic acid |
|  | 3α-hydroxyandrostan-4-en-17β-one | 3α-hydroxy-5α-androstan-17-one | androst-4-en-3,17-dione | 5α-androstane-3,17-dione |
|  | 5β-androstane-3,17-dione | 5β-androstane-3α,17β-diol | 5α-androstane-3β,17α-diol | valine |
|  | muscopyridine | muscone | hydroxymuscopyridine B | 3β- hydroxy-5β-androstan-17-one |
|  | 3α- hydroxy-5β-androstan-17-one | 3β- hydroxy-androst-5-en-17-one | 5α-androstane-3,17-diol | 5β-androstane-3α,17α-diol |
|  | 3β-hydroxyandrost-5-en-17-one |  |  |  |
| Renshen | kaempferol | Celabenzine | Deoxyharringtonine | Dianthramine |
|  | Frutinone A | Girinimbin | Gomisin B | malkangunin |
|  | Panaxadiol | suchilactone | Fumarine |  |
| Niuhuang | aspartate | deoxycholic acid | glycine | alanine |
|  | methionine | cholic acid | leucine |  |
| Rougui | cinnamyl acetate | syringaresinol | 4'-O-methyl-(+)-catechin | 3'-O-methyl-(-)-epicatechin |
|  | cinncassiol A | phenylmethylbenzoate | benzaldehyde | coumarin |
|  | cinnamic acid | ethyl cinnamate | cinnamic alcohol | cinnamaldehyde |
|  | protocatechuic acid | cinnzeylanine | cinnzeylanol | cinncassiol C1 |
|  | cinncassiol C2 | cinncassiol C3 | cinncassiol D1 | cinncassiol D2 |
|  | cinncassiol D3 | cinncassiol D4 | cinncassiol D1 |  |
| Suhexiang | linalool | styracin | benzoic acid | 4-terpineol |
|  | α-terpineol | cinnamic acid | methyl cinnamate | cinnamaldehyde |
|  | cineole | allylphenol | 4-terpineol | α-terpineol |
|  | ethylphenol | dihydrocoumarone | β-phenylpropionic acid | n-propyl cinnamate |
| Chansu | gamabufotalin | cinobufotalin | cinobufagin | cinobufaginol |
|  | Q-bufarenogin | telocinobufagin | arenobufagin | epinephrine |
|  | marinobufagin | resibufogenin | resibufagin | dehydrobufotenine |
|  | 5-hydroxytryptamine | 7α-hydroxycholesterol | 7β-hydroxycholesterol | helleprigenin |
|  | bufotalin | bufalin | bufotenine | bufotenidine |
|  | bufothionine |  |  |  |
| Bingpianq | asiatic acid | bronyl acetate | dipterocarpol |  |
