# Supplementary material for: Shexiang Baoxin Pills Inhibited Proliferation and Migration of Human Coronary Artery Smooth Muscle Cells via PI3K/AKT/mTOR Pathway
Source: Front Cardiovasc Med. 2021 Aug 25;8:700630. doi: 10.3389/fcvm.2021.700630 (PMC8425485; doi:10.3389/fcvm.2021.700630)
Supplement: Supplementary file 3 [file Table_3.docx]

Table S3 The potential pathways of SBP on CAD.

| ID | Description | *P* value | *P* .adjust |
| --- | --- | --- | --- |
| hsa04151 | PI3K-Akt signaling pathway | 1.18E-05 | 6.15E-05 |
| hsa04660 | T cell receptor signaling pathway | 1.30E-05 | 6.62E-05 |
| hsa05206 | MicroRNAs in cancer | 1.37E-05 | 6.88E-05 |
| hsa05169 | Epstein-Barr virus infection | 1.57E-05 | 7.76E-05 |
| hsa05132 | Salmonella infection | 2.00E-05 | 9.68E-05 |
| hsa04066 | HIF-1 signaling pathway | 2.10E-05 | 9.97E-05 |
| hsa05212 | Pancreatic cancer | 2.18E-05 | 0.000102 |
| hsa00140 | Steroid hormone biosynthesis | 2.22E-05 | 0.000102 |
| hsa04380 | Osteoclast differentiation | 2.28E-05 | 0.000103 |
| hsa04926 | Relaxin signaling pathway | 2.48E-05 | 0.00011 |
| hsa04750 | Inflammatory mediator regulation of TRP channels | 3.88E-05 | 0.00017 |
| hsa04913 | Ovarian steroidogenesis | 4.06E-05 | 0.000175 |
| hsa04630 | JAK-STAT signaling pathway | 6.76E-05 | 0.000284 |
| hsa04622 | RIG-I-like receptor signaling pathway | 6.82E-05 | 0.000284 |
| hsa05235 | PD-L1 expression and PD-1 checkpoint pathway in cancer | 8.69E-05 | 0.000357 |
| hsa04014 | Ras signaling pathway | 9.20E-05 | 0.000368 |
| hsa05134 | Legionellosis | 9.24E-05 | 0.000368 |
| hsa04015 | Rap1 signaling pathway | 9.40E-05 | 0.000369 |
| hsa04370 | VEGF signaling pathway | 0.000119 | 0.000457 |
| hsa04932 | Non-alcoholic fatty liver disease | 0.00012 | 0.000457 |
