# Supplementary figures and images for: Shexiang Baoxin Pills Inhibited Proliferation and Migration of Human Coronary Artery Smooth Muscle Cells via PI3K/AKT/mTOR Pathway
Source: Front Cardiovasc Med. 2021 Aug 25;8:700630. doi: 10.3389/fcvm.2021.700630 (PMC8425485; doi:10.3389/fcvm.2021.700630)

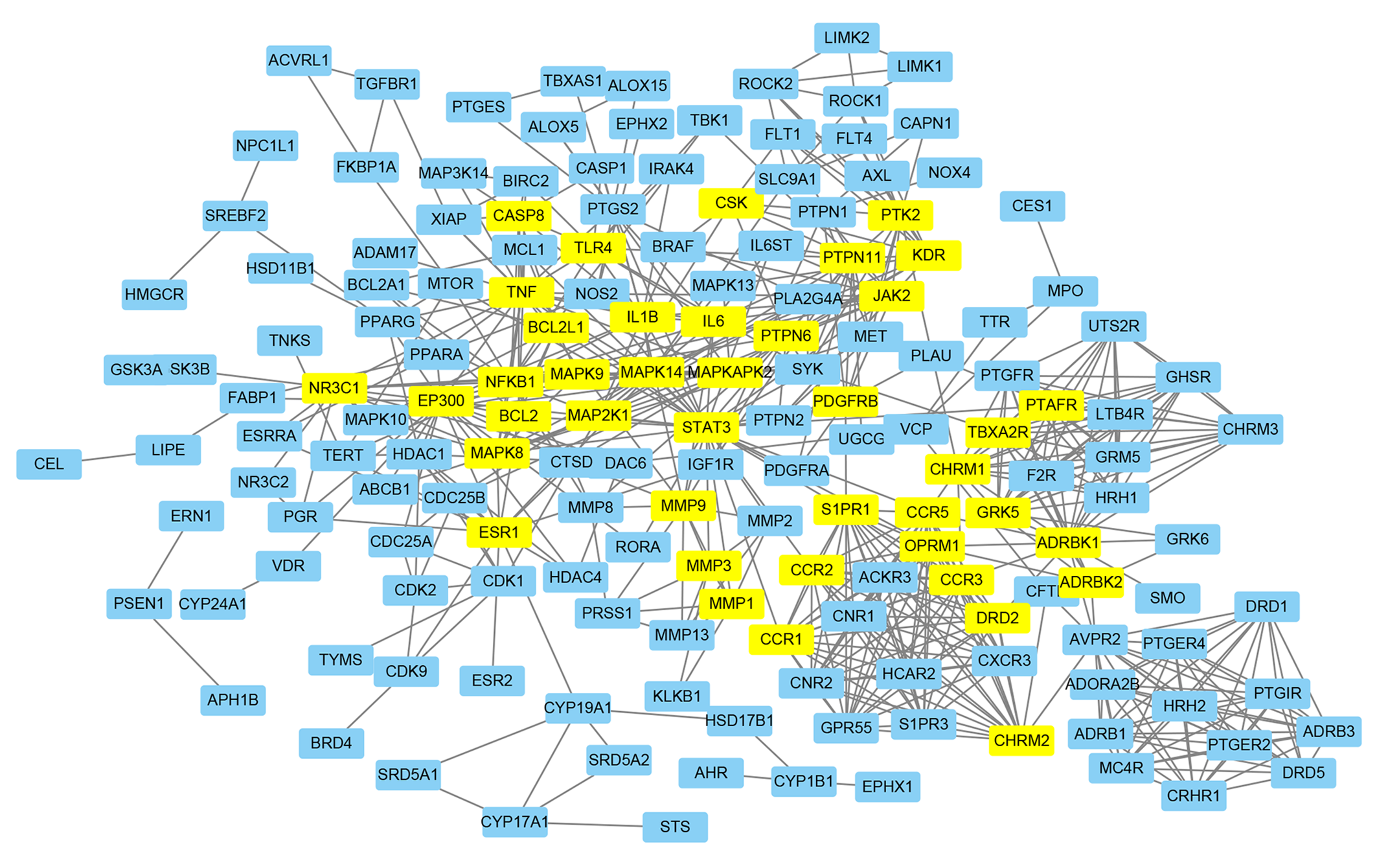

Supplement: Supplementary file 4 [file Image_1.TIF]

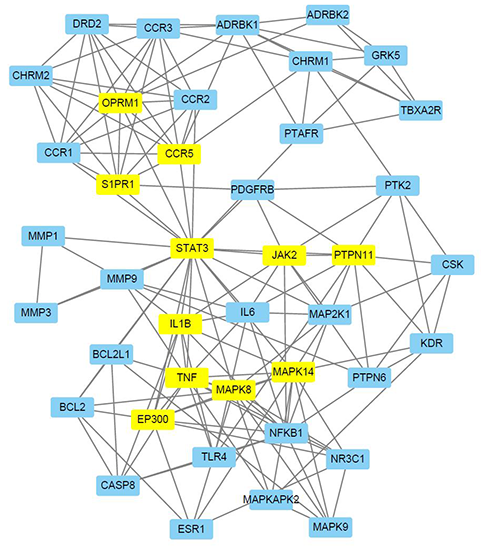

Supplement: Supplementary file 5 [file Image_2.TIF]

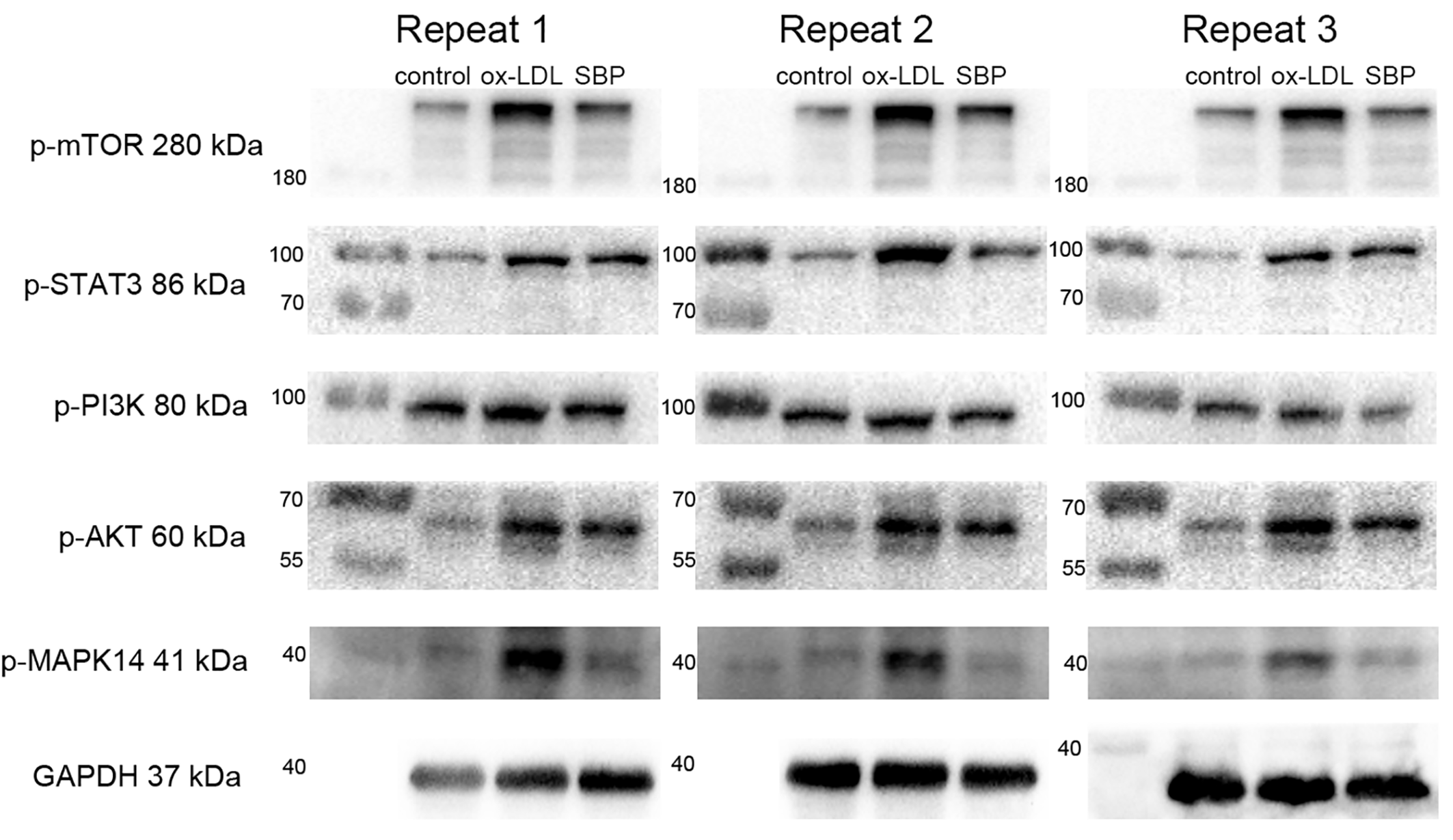

Supplement: Supplementary file 6 [file Image_3.TIF]
